# Treatment of Hydrocele with Ioduretted Injections

**Published:** 1845-02

**Authors:** 


*Treatment of Hydrocele with Ioduretted Injections.*—In more than three hundred cases of this complaint treated with an ioduretted injection, (composed of tincture of iodine four parts, and distilled water one hundred and twenty-five parts,) by M. Velpeau, not a single accident or unpleasant symptom has ever occurred. One of the patients, indeed, died; but the fatal result in this instance proceeded from a purulent inflammation of the cellular tissue of the pelvis, quite unconnected with the operation, and not having any communication whatever with the affection of the scrotum. The average period for effecting the cure was fifteen days. In one case only the injection found its way into the tissue of the scrotum, in place of the tunica vaginalis; notwithstanding this misadventure, no appearance of gangrene supervened, and the patient recovered without any unpleasant accident.

*Med. Chir. Rev.*, from *L'Experience.*

